# Technical nuances for the resection of cervical dumbbell schwannomas

**DOI:** 10.3171/2023.7.FOCVID2361

**Published:** 2023-10-01

**Authors:** Brandon M. Wilkinson, Disep I. Ojukwu, Timothy Dawson, Cheerag Upadhyaya, Michael A. Galgano

**Affiliations:** 1 Department of Neurosurgery, SUNY Upstate Medical University, Syracuse, New York;; 2 St. George’s University, School of Medicine, Great River, New York; and; 3 Department of Neurosurgery, University of North Carolina, Chapel Hill, North Carolina

**Keywords:** cervical schwannomas, intradural tumors, intraoperative surgical video, neurosurgery, spine

## Abstract

The majority of spinal nerve sheath tumors are within the intradural/extramedullary compartment. A subset of these tumors develop extraforaminal components that gradually expand into potential spaces. Herein, the authors provide a 2D video demonstrating the technical nuances concerning resection of cervical dumbbell schwannomas with extraspinal extension. Although nerve sheath tumors with large extraforaminal extension are often associated with complications and pose unique challenges to surgeons, circumferential exposure with intradural exploration allows for gross-total resection and nerve root preservation, without need for adjuvant treatments. The use of intraoperative ultrasound, neurophysiological monitoring, Doppler imaging, and meticulous surgical techniques aided to circumvent complications.

## Transcript

### 0:27 History and Physical.

This operative video will illustrate the technical nuances for resection of a cervical dumbbell schwannoma. The patient is a 23-year-old female presenting with progressive gait imbalance and left hand and triceps weakness.

### 0:33 Preoperative Imaging.

Preoperative MRI scan reveals a homogeneously enhancing lesion causing severe spinal cord compression emanating into the left-sided C8 neuroforamen.^1^ Preoperative CAT scan reveals associated bony remodeling.^2^

### 0:48 Surgical Plan.

The surgical plan is as follows.

### 0:52 Removal of Intradural Component of Tumor.

After performing standard subperiosteal dissection, we placed lateral mass screws at C4, C5, and C6. We then performed freehand thoracic pedicle screws using the funnel technique. Here, we drill the outer cortex of the pedicle, followed by gear shift, then subsequently freehand screw placement. After the thoracic pedicle screws were placed, we then performed an en bloc laminectomy at C6, C7, and T1. We then performed a complete facetectomy on the left at C7/T1 for exposure of the extradural component of the tumor. We also performed an inferior facetectomy on the left at C6/7. This aided mobilization and exposure of the C7 nerve root. We then cauterized the capsule of the tumor for further mobilization and shrinkage. Here, the C7 nerve root can clearly be seen.^3^ We then brought in the ultrasound to confirm that there was, in fact, an intradural component of the tumor.^1,4^ Once this was confirmed, we performed a midline durotomy using an 11 blade. 5-0 Nurolon dural tack-ups were then utilized. Neuromonitoring then confirmed that there were no motor branches entering the tumor.^5,6^ Because these were felt to be dorsal sensory branches, they were cauterized and divided. Here, we can clearly see the motor root of C8 on the left side. We then closed the inner durotomy on the lateral side with 5-0 Nurolons and 6-0 Prolenes. After the lateral durotomy was closed, we then paid our attention to closure of the midline durotomy using 5-0 Nurolons.

### 3:08 Removal of Extradural Component of Tumor.

We then brought our attention back to the extradural component of the tumor. It was much easier to mobilize now that the intradural component had been resected. We subsequently performed further cauterization of the capsule for mobilization. We can start to see the scalloped C7 vertebral body here. We then performed internal debulking of the tumor to aid in further mobilization. The ventral and lateral components of the tumor were carefully dissected. The remaining attachments were then cut. Final tumor delivery can be seen here. We then brought a Doppler in to confirm pulsatile flow of the left side of vertebral artery.^7^ Interconnecting rods were then placed and final locked.

### 4:10 Postoperative Imaging.

Postoperative imaging reveals a gross-total resection of the tumor. Hardware position was confirmed with a postoperative CAT scan. Adequate sagittal and coronal cervical balance can be appreciated.

### 4:24 Postoperative Course.

The patient had an uneventful postoperative course. She was eventually discharged to inpatient acute rehabilitation for 1 week. At 2-year follow-up, she has continued motor strength improvement in her left upper extremity and lower extremity with resolution of her myelopathic symptoms.

## Acknowledgments

We sincerely thank Xian M. Boles, 3D Medical Illustrator at the University of North Carolina Department of Neurosurgery, for the exquisite and skillful rendition of our case.

## Disclosures

The authors report no conflict of interest concerning the materials or methods used in this study or the findings specified in this publication.

## Author Contributions

Primary surgeon: Wilkinson, Galgano. Editing and drafting the video and abstract: all authors. Critically revising the work: all authors. Reviewed submitted version of the work: all authors. Approved the final version of the work on behalf of all authors: Ojukwu. Supervision: Galgano.

## Supplemental Information

Supplemental Fig. 1

## Patient Informed Consent

The necessary patient informed consent was obtained in this study.
