# Supplementary material for: Technical nuances for the resection of cervical dumbbell schwannomas
Source: Neurosurg Focus Video. 2023 Oct 1;9(2):V14. doi: 10.3171/2023.7.FOCVID2361 (PMC10580742; doi:10.3171/2023.7.FOCVID2361)
Supplement: Supplemental Fig. 1 [file SupplementalFig1_FOCVID23-61.pdf]

NEUROSURGICAL FOCUS VIDEO

ONLINE ONLY

Supplemental material

Technical nuances for the resection of cervical dumbbell schwannomas

Wilkinson et al.

https://thejns.org/doi/abs/10.3171/2023.7.FOCVID2361

DISCLAIMER The *Journal of Neurosurgery* acknowledges that the following section is published verbatim as submitted by the authors and did not go through either the *Journal's* peer-review or editing process.

© 2023 The authors, CC BY 4.0 (http://creativecommons.org/licenses/by/4.0/)

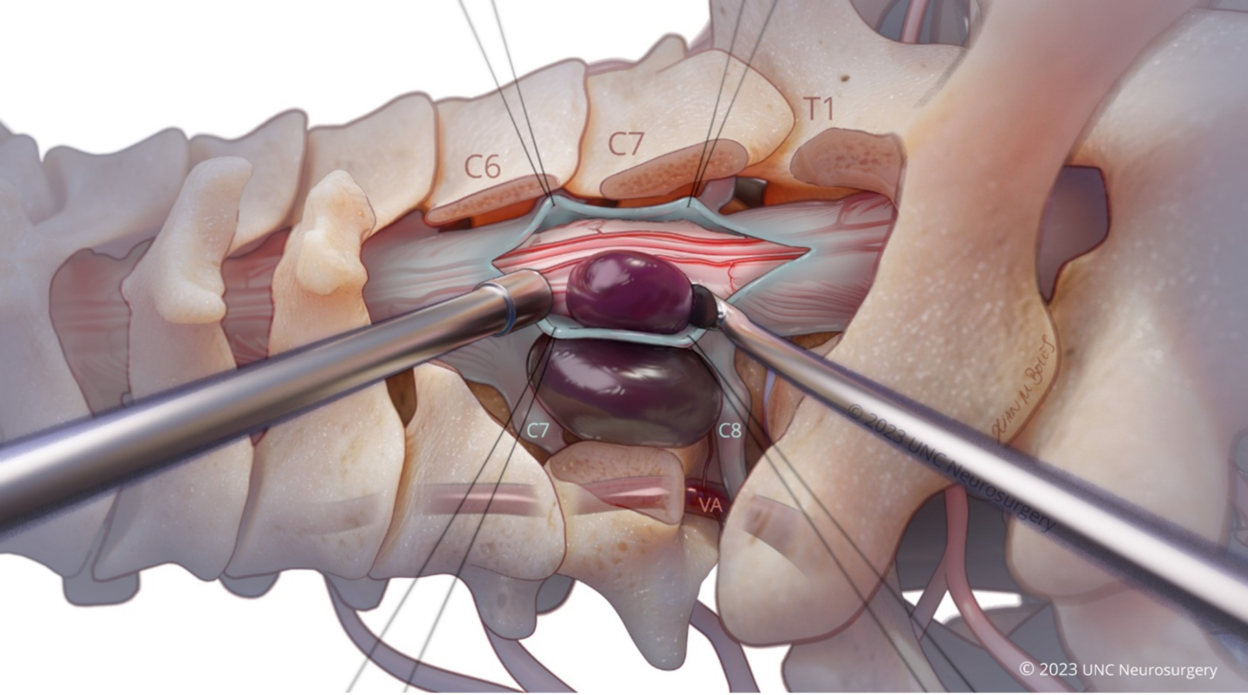

Supplemental Figure 1. Illustration of intraoperative gross total resection of a C8 dumbbell schwannoma with extraspinal extension. Although intradural and extradural components of the lesion caused significant spinal cord compression, complete resection and nerve root preservation were successfully achieved. Used with permission of University of North Carolina, Department of Neurosurgery. All rights reserved.
